# Supplementary material for: Quantum annealing of a frustrated magnet
Source: Nat Commun. 2024 Apr 25;15:3495. doi: 10.1038/s41467-024-47819-y (PMC11045780; doi:10.1038/s41467-024-47819-y)
Supplement: Supplementary file 1 — Supplementary Information for “Quantum annealing of a frustrated magnet” [file 41467_2024_47819_MOESM1_ESM.pdf]

Supplementary Information

Quantum annealing of a frustrated magnet

Yuqian Zhao, Zhaohua Ma, Zhangzhen He, Haijun Liao, Yan-Cheng Wang, Junfeng Wang & Yuesheng Li

Supplementary Note 1. Principal axes of the effective Ising spin system of α-CoV_2O_6

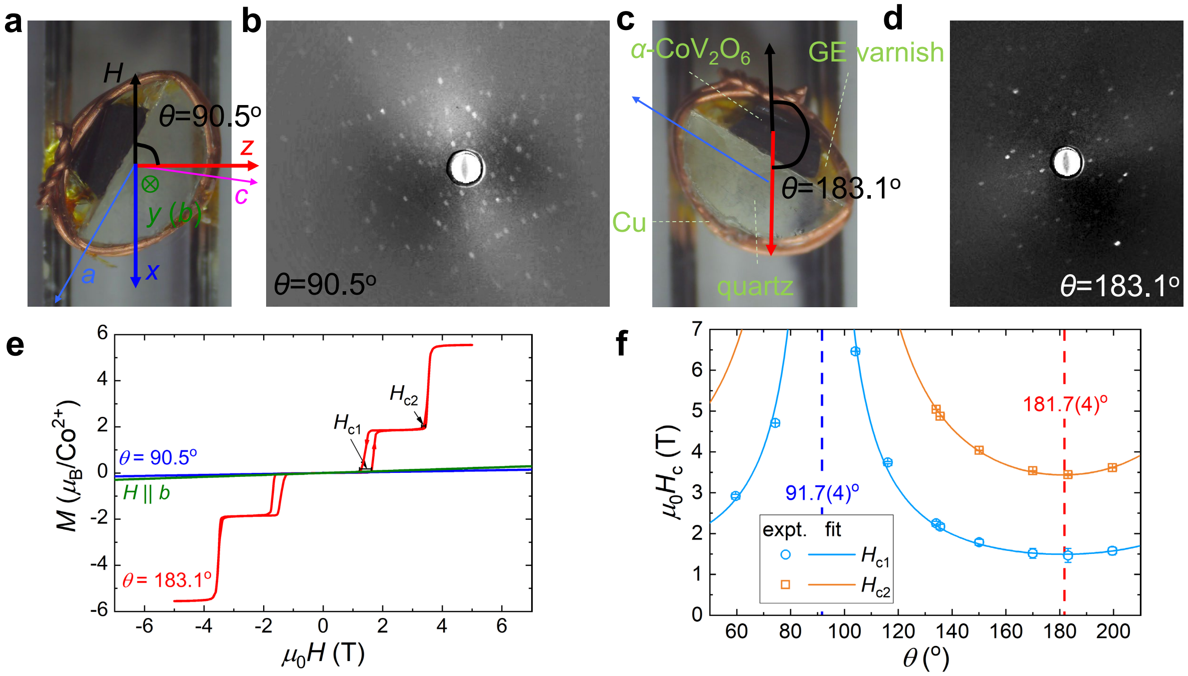

Supplementary Fig. 1 | Magnetization measured on the single crystal of α-CoV_2O_6. a, **c** Images of the crystal at rotation angles, θ = 90.5° and 183.1°, respectively. **b**, **d** The rotation angles were determined by Laue x-ray diffraction measurements and further confirmed with microscopic images (e.g., **a**, **c**), with a maximum error of $\sim \pm 1°$. **e** Magnetization measured at θ = 90.5° and 183.1° and at 5 K. The critical fields (H_{c1} and H_{c2}) are indicated, and the magnetization measured along the b axis is displayed for comparison. **f** Rotation-angle dependence of H_{c1} and H_{c2} measured at 5 K. The solid lines represent the combined fit to the experimental data using Supplementary Eq. (1), and the dashed blue and red lines show the positions of the other two principal axes as determined by the fit, with $H_{c1} = \infty$ ($H_{c2} = \infty$) and $H_{c1} = H_{c1}^z$ ($H_{c2} = H_{c2}^z$), respectively. Error bars, 1σ s.e.

High-quality single crystals of α-CoV_2O_6 (space group: $C121$) were grown using the flux method [1–3]. The nonmagnetic reference compound α-ZnV_2O_6 used to measure the lattice specific heat was synthesized through a traditional solid-phase method using stoichiometric mixtures of $ZnC_2O_4{\cdot}2H_2O$ and V_2O_5 in air up to 630 °C for 60 hours, with intermediate grinding. The phase purity of the sample was verified by

x-ray diffraction. Due to the strong (Ising) anisotropy of the magnetism of the α-CoV_2O_6 spin system [4, 5], the single crystals were cut to the appropriate size in our measurements to maintain a high signal-to-noise ratio while avoiding excessive stress. For all magnetization measurements in a magnetic properties measurement system (MPMS) up to 7 T, we selected a single crystal weighing 10.52 mg with a natural surface corresponding to the ab plane (0 0 1), as confirmed by Laue x-ray diffraction. The crystal was affixed to a quartz sample holder with GE varnish and cooper wire (Cu $\geq$ 99.999%), as depicted in Supplementary Fig. 1a, c. We verified the orientation and position of the crystal following each MPMS measurement, and no movement relative to the sample holder was detected. In α-CoV_2O_6, the two-fold rotation axis is oriented along the b direction, indicating that b must be one of the three principal axes (i.e., the y axis), while the other two principal axes are orthogonal to the b axis. Supplementary Fig. 1e displays the magnetization measured along the b axis, which exhibits a linear field dependence at least up to 7 T, consistent with the findings reported in previous references [1, 3]. This observation confirms the designation of b as one of the principal axes.

Lenertz et al. reported that at low temperatures the magnetic moments (i.e., Ising spins) of Co^{2+} in α-CoV_2O_6 are aligned along the CoO_6 octahedra axis (z, which is perpendicular to b), as determined by neutron diffraction measurements performed under various applied magnetic fields [4]. To confirm this, we performed magnetization measurements perpendicular to the b axis by rotating the crystal through an angle θ, as illustrated in Supplementary Fig. 1. Here, θ is the angle between the z axis (i.e., the Ising axis) and the direction of the applied magnetic field ($\mathbf{H}$). At 5 K, we observed two critical fields, $H_{\rm c1}$ and $H_{\rm c2}$, consistent with previous reports in Ref. [1]. These critical fields are associated with the successive transitions among the three phases characterized by M^z = 0 (interchain antiferromagnetic), g^z/6 (1/3-plateau), and g^z/2 (fully polarized), respectively [4, 5]. The dependence of these critical fields on θ was calculated for the crystal of α-CoV_2O_6, as

$$H_{\rm c1} = \frac{H^z_{\rm c1}}{|\cos(\theta - \theta_0)|}, \quad H_{\rm c2} = \frac{H^z_{\rm c2}}{|\cos(\theta - \theta_0)|}, \tag{1}$$

where $\mu_0 H^z_{\rm c1} \sim$ 1.49 T and $\mu_0 H^z_{\rm c2} \sim$ 3.44 T are the minimum values of $\mu_0 H_{\rm c1}$ and $\mu_0 H_{\rm c2}$, respectively, and θ_0 represents the offset of θ. Our combined fit to the experimental data yielded a small offset angle of θ_0 = 1.7±0.4° (see Supplementary Fig. 1f), consistent with the previous neutron diffraction work [4].

For the effective Ising spin system of α-CoV_2O_6, we define a rectangular coordinate system with the principal axes, x, y, and z, as shown in Supplementary Fig. 1a and Supplementary Fig. 5a.

Supplementary Note 2. Crystal-field Hamiltonian with spin-orbit coupling in α-CoV_2O_6

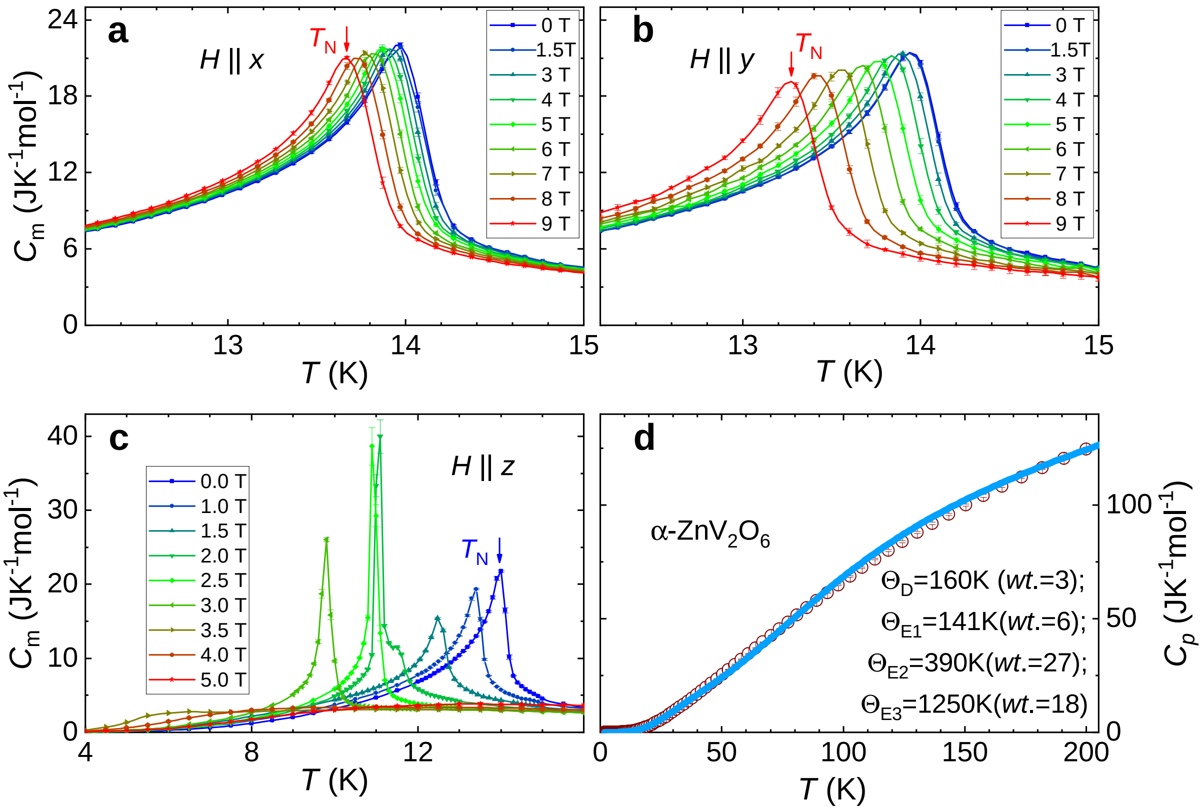

Supplementary Fig. 2 | **Specific heat of α-CoV$_2$O$_6$ and α-ZnV$_2$O$_6$. a-c** Magnetic specific heat measured on α-CoV$_2$O$_6$ under applied magnetic fields along the x, y, z axes, respectively. **d** The lattice contribution measured on α-ZnV$_2$O$_6$ is subtracted in **a-c**. The colored line represents a Debye-Einstein fit which yields the Debye temperature Θ_D = 160 K. Error bars, 1σ s.e.

We investigate the single-ion interactions in α-CoV$_2$O$_6$ to understand the relationship between the applied transverse magnetic field $\mu_0 H^\perp$ (in T) and the resulted transverse field $\mathit{\Gamma}$ (in K). The crystalline electric field (CEF) of Co^{2+} is primarily determined by the local CoO$_6$ environment [6]. The CoO$_6$ octahedra possess approximate C_{2v} point-group symmetry (see Supplementary Fig. 5a). The crystal structure of α-CoV$_2$O$_6$ is two-fold rotation symmetrical about the y axis, and the dihedral angle of O1–Co-Co–O2 (with Co-Co along the y axis) is 90.04° close to a right angle [1], resulting in good mirror symmetries on both xy and yz planes for the CoO$_6$ octahedra. Therefore, we start with the single-ion Hamiltonian that is invariant under the C_{2v} point-group symmetry [7],

$$\mathcal{H}_\mathrm{SI} = B_2^0 O_2^0 + B_2^2 O_2^2 + B_4^0 O_4^0 + B_4^2 O_4^2 + B_4^4 O_4^4 + B_6^0 O_6^0 + B_6^2 O_6^2 + +B_6^4 O_6^4 + B_6^6 O_6^6 + \lambda \mathbf{s}\cdot\mathbf{L} - \mu_0 \mu_B \mathbf{H}\cdot(2\mathbf{s}+\mathbf{L}), \quad (2)$$

where B_n^m (m, n are integers and $m \leq n$) are CEF parameters, O_n^m are the Stevens operators, and λ = -22.32 meV the spin-orbit (**s**-**L**) coupling [8, 9]. For Co^{2+}, s = 3/2 and L = 3 give rise to a $(2s+1)(2L+1)$ = 28 dimensional Hilbert space of $\mathcal{H}_{\mathrm{SI}}$. By diagonalizing $\mathcal{H}_{\mathrm{SI}}$, we obtain the eigenvalues and eigenvectors, E_j and $|E_j\rangle$ (j = 1, ..., 28), respectively. The single-ion dc magnetic susceptibility is calculated by,

$$\chi_{\mathrm{SI}}^{\alpha} = \frac{N_{\mathrm{A}}\mu_{\mathrm{B}}\sum_{j=1}^{28}\exp(-\frac{E_j}{k_{\mathrm{B}}T})\langle E_j|2s^{\alpha} + L^{\alpha}|E_j\rangle}{H^{\alpha}\sum_{j=1}^{28}\exp(-\frac{E_j}{k_{\mathrm{B}}T})}, \tag{3}$$

where α = x', y', z' in the CEF coordinate system with x' = x, y' = z, z' = $-y$ (see Supplementary Fig. 5a for the definition of the xyz coordinate system). It is noteworthy that the small transverse susceptibilities (measured with $H \parallel x$ and y, see Supplementary Fig. 3) originate from the Van Vleck paramagnetism and can be well understood in principle through the single-ion Hamiltonian. At zero applied field $\mathbf{H}$ = (0, 0, 0), 14 CEF doublets of the eigenvectors form with E_1 = E_2, ..., E_{27} = E_{28}, which are protected by the time-reversal symmetry of the system in accordance with Kramers' law. In the subspace of the ground-state CEF doublet, denoted by $|E_1\rangle$ and $|E_2\rangle$, the matrix element of the magnetic moment can be expressed as $m_{jj'}^{\alpha}$ = $\langle E_j|2s^{\alpha} + L^{\alpha}|E_{j'}\rangle$, j, j' = 1, 2. Diagonalizing the matrix $\mathbf{m}^{\alpha}$ yields two eigenvalues, $-g^{\alpha}/2$ and $g^{\alpha}/2$, from which the g factor g^{α} can be obtained.

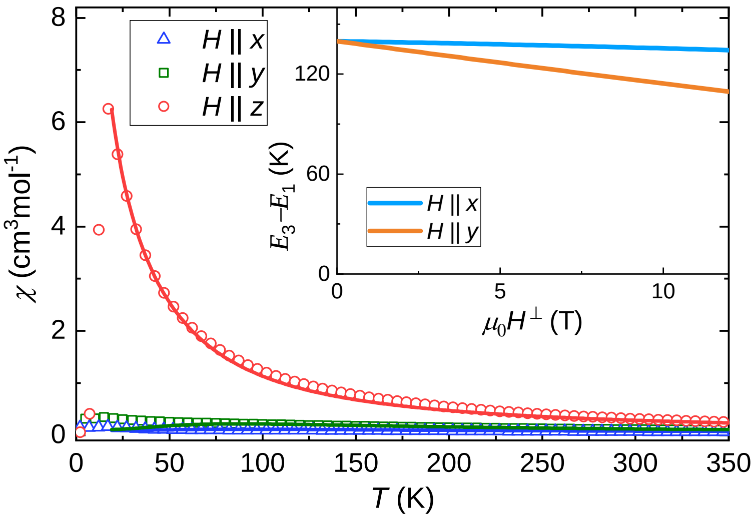

Supplementary Fig. 3 | Fit to magnetic susceptibilities with tuned single-ion crystal-field parameters. Temperature dependence of the susceptibilities measured at 1 T applied along the x, y, z axes. The colored lines show the least-square fit above $T_{\mathrm{N}} \sim$ 14 K using the single-ion Hamiltonian of Supplementary Eq. (2). The inset presents the calculated energy gaps between the second-excited and ground-state single-ion states, $E_3 - E_1$, in transverse magnetic fields applied along the x and y axes.

From least-squares fitting the experimental magnetic susceptibilities (see Supplementary Fig. 3) and g factors (with $g_{\mathrm{e}}^{x'}$ = g^x = 0, $g_{\mathrm{e}}^{y'}$ = g^z = 11.04, $g_{\mathrm{e}}^{z'}$ = g^y = 0), we obtain B_2^0 = 2.50 meV, B_2^2 = 3.14 meV, B_4^0 =

-0.336 meV, $B_4^2 = -1.22$ meV, $B_4^4 = -2.61$ meV, $B_6^0 = -0.00266$ meV, $B_6^2 = 0.191$ meV, $B_6^4 = 0.340$ meV, $B_6^6 = 0.578$ meV, and the calculated g factors, $g_c^{x'} = 0.0$, $g_c^{y'} = 11.04 \sim g^z$, $g_c^{z'} = 0.0$. At low temperatures and low applied fields, the magnetism of α-CoV_2O_6 is primarily determined by the two lowest-lying single-ion states ($|E_1\rangle$ and $|E_2\rangle$), which are well-separated from other excited CEF states with an energy gap of $E_3 - E_1 \sim 140$ K (see inset of Supplementary Fig. 3).

At non-zero applied field, the ground-state CEF Kramers doublet is lifted with $E_2 > E_1$, due to the breaking of time-reversal symmetry. When a magnetic field is applied along the Ising (z) axis, the single-ion Hamiltonian of Supplementary Eq. (2) is represented as the well-known Zeeman term $\mathcal{H}_{\rm SI} = -\mu_0\mu_{\rm B}H^z g^z S^z + C$ in the subspace of $|E_1\rangle = |S^z = S\rangle$ and $|E_2\rangle = |S^z = -S\rangle$, where $E_2 - E_1 = \mu_0\mu_{\rm B}H^z g^z$, $C = (E_1 + E_2)/2$, $S = 1/2$, and

$$\mathbf{m}^x \sim \mathbf{m}^y \sim \begin{pmatrix} 0 & 0 \\ 0 & 0 \end{pmatrix}, \mathbf{m}^z \sim \frac{g^z}{2}\begin{pmatrix} 1 & 0 \\ 0 & -1 \end{pmatrix} = g^z S^z. \quad (4)$$

When the magnetic field is applied along the transverse (x or y) axis, in the subspace of $|E_1\rangle$ and $|E_2\rangle$ the magnetic moment is presented as

$$\mathbf{m}^x \sim \mathbf{m}^y \sim \begin{pmatrix} 0 & 0 \\ 0 & 0 \end{pmatrix}, \mathbf{m}^z \sim \frac{g^z}{2}\begin{pmatrix} 0 & -i \\ i & 0 \end{pmatrix} = g^z S^y. \quad (5)$$

The eigenstates of Supplementary Eq. (5) are also the effective spin-1/2 ($S = 1/2$) states,

$$|S^z = \pm S\rangle = \frac{1}{\sqrt{2}}(|E_1\rangle \pm i|E_2\rangle) \quad (6)$$

The single-ion Hamiltonian of Supplementary Eq. (2) can be expressed as the well-known transverse-field term $\mathcal{H}_{\rm SI} = (E_2 - E_1)(|E_2\rangle\langle E_2| - |E_1\rangle\langle E_1|)/2 + C = -\mathit{\Gamma} S^x + C$, in the subspace of the effective S = 1/2 states [10, 11]. Here, $\mathit{\Gamma} = E_2 - E_1$ is the transverse field, and $S^x = S(|S^z = S\rangle\langle S^z = -S| + |S^z = -S\rangle\langle S^z = S|) = -(|E_2\rangle\langle E_2| - |E_1\rangle\langle E_1|)/2$ is the effective S = 1/2 operator.

Supplementary Note 3. Classical Monte Carlo simulations at zero transverse field

The magnetic structures of α-CoV_2O_6 at low temperatures and various applied magnetic fields were extensively studied using neutron diffraction measurements [4]. Saúl reported a comprehensive study of the magnetic interactions using density functional theory (DFT) and Monte Carlo (MC) calculations [5], which exhibited qualitative agreement with the experimentally observed magnetic structures. However, the thermodynamic properties of α-CoV_2O_6 have not been fully reproduced and the Hamiltonian parameters are still not fully refined. This is due to the presence of complex metastable states below ~ 6 K, as indicated

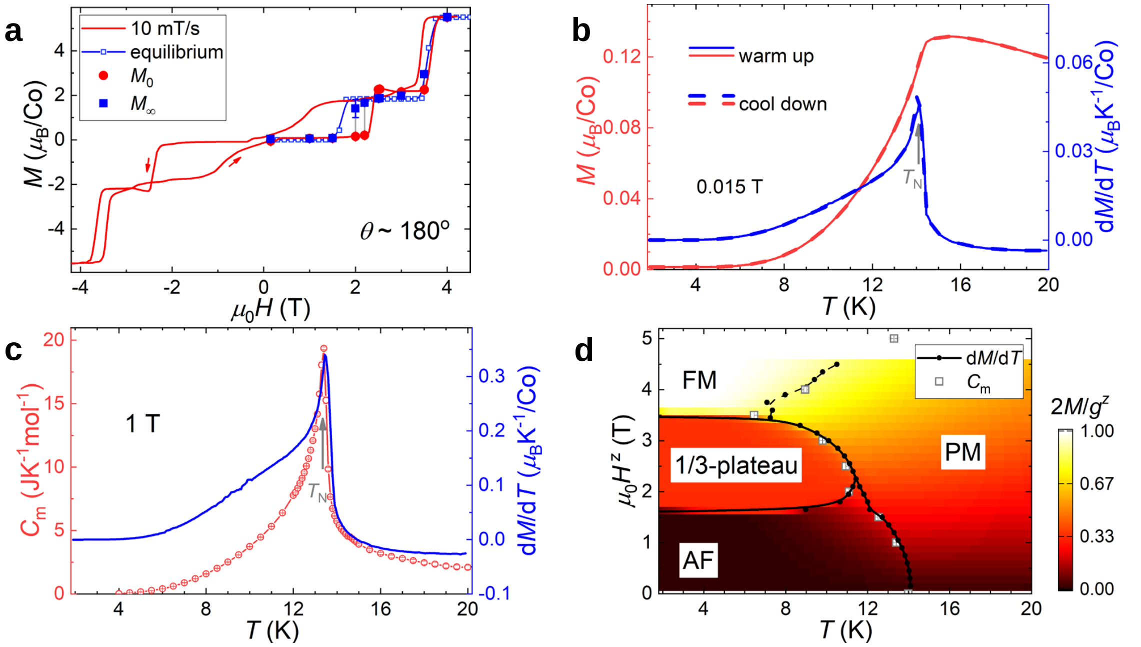

Supplementary Fig. 4 | Quasi-equilibrium-state magnetism via thermal annealing. a Magnetization (M) of α-CoV_2O_6 measured with the magnetic field applied along the z (Ising) axis at 1.9 K. The red lines represent the hysteresis loop measured at a constant ramp rate of 10 mT/s, with the blue line showing the quasi-equilibrium-state M extracted from **d**. M_0 (red scatters) and M_∞ (blue scatters) are the magnetization at t = 0 and ∞ obtained from fitting the relaxation curves (see Supplementary Fig. 8b for example). **b** Temperature dependence of quasi-equilibrium M and dM/dH measured at 15 mT. After achieving the steady field at 1.9 K (t = 0), we waited for 30 min before measuring M versus T by warming up to 20 K and subsequently cooling back down to 1.9 K. **c** Temperature dependence of quasi-equilibrium specific heat and dM/dH measured at 1 T. The critical temperatures T_N are indicated in **b** and **c**. **d** Quasi-equilibrium M measured by sweeping temperature at steady fields after allowing sufficient time for relaxation. The phase boundaries are determined based on the quasi-equilibrium dM/dH and specific heat measurements (see Supplementary Fig. 2c), and the different phases, including interchain antiferromagnetic (AF), up-up-down 1/3-plateau, fully-polarized ferromagnetic (FM), and high-T paramagnetic (PM) phases, are indicated. Error bars, 1σ s.e.

by the observed magnetic hysteresis (see Supplementary Fig. 4a) [3, 12]. To obtain quasi-equilibrium-state thermodynamic data, we allowed the spin system to fully relax for a time t longer than the intrinsic relaxation time τ (i.e., thermal annealing time), which is commonly less than ~1,000 s above $\sim$ 1.9 K (see Supplementary Fig. 8). The magnetization M, measured after waiting 30 min for the system to relax, shows no evident magnetic hysteresis (see Supplementary Fig. 4b), and the dM/dH and specific heat C_m only exhibit one sharp peak (see Supplementary Fig. 4b, c), consistent with theoretical predictions of equilibrium-state properties [5]. This confirms the approach to quasi thermodynamic equilibrium in our

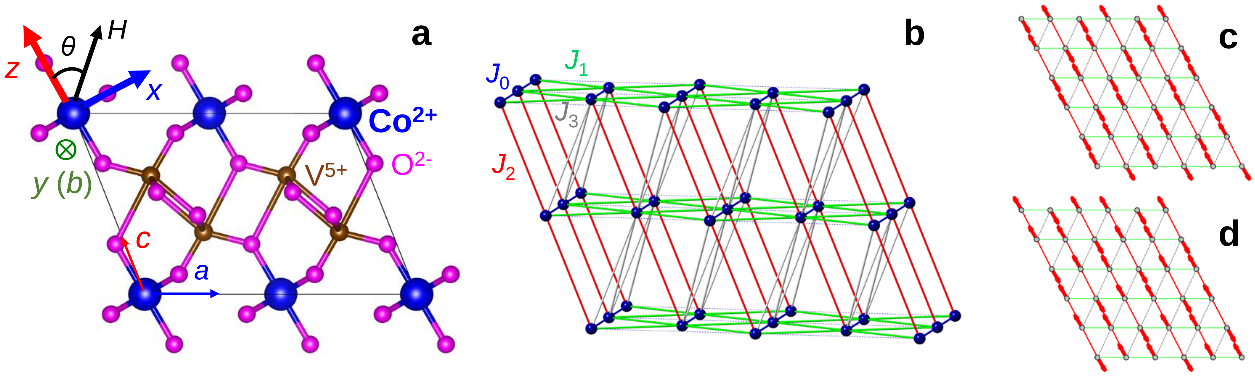

Supplementary Fig. 5 | Effective spin-1/2 interactions and magnetic structures in α-CoV_2O_6. a Crystal structure of α-CoV_2O_6, viewed along the b axis. The thin lines show the unit cell and the coordinate system for the spin components is established. **b** First (J_0, $|\text{Co-Co}|_0 \sim 3.5$ Å), second (J_1, $|\text{Co-Co}|_1 \sim 4.9$ Å), third (J_2, $|\text{Co-Co}|_2 \sim 6.6$ Å), and fourth (J_3, $|\text{Co-Co}|_3 \sim 6.8$ Å) nearest neighbor Ising interactions. **c** The stripe antiferromagnetic state at $|H^z| < H^z_{\text{c1}}$. **d** The one-third magnetization plateau state at $H^z_{\text{c1}} < |H^z| < H^z_{\text{c2}}$.

measurements.

Supplementary Table 1 | Ising spin Hamiltonian parameters of α-CoV_2O_6. Previously reported theoretical parameters (no. 1) [5] and those obtained by fitting the quasi-equilibrium-state magnetization (no. 2, see Supplementary Fig. 6a). The standard deviation σ_d is defined using the experimental magnetization M^e_k, calculated magnetization M^c_k, and the number of data points N.

interactions	no. 1 [5]	no. 2 (this work)
intrachain J_0	-29.8 K	-30.73 K
interchain J_1	4.9 K	3.60 K
interchain J_2	10.6 K	14.21 K
interchain J_3	2 K	2.55 K
$\sigma_\text{d} = \sqrt{\sum_k (M^\text{e}_k - M^\text{c}_k)^2/N}$	0.47 μ_B/Co	0.08 μ_B/Co

In this work, we start from the previously reported spin-1/2 Hamiltonian in zero transverse field (by setting $\mathit{\Gamma} = 0$ K, see below) [5] (see Supplementary Fig. 5b),

$$\mathcal{H} = J_0 \sum_{\langle i,i_0\rangle} S^z_i S^z_{i_0} + J_1 \sum_{\langle i,i_1\rangle} S^z_i S^z_{i_1} + J_2 \sum_{\langle i,i_2\rangle} S^z_i S^z_{i_2} + J_3 \sum_{\langle i,i_3\rangle} S^z_i S^z_{i_3} - \mu_0\mu_\text{B} H^z g^z \sum_i S^z_i - \mathit{\Gamma} \sum_i S^x_i . \quad (7)$$

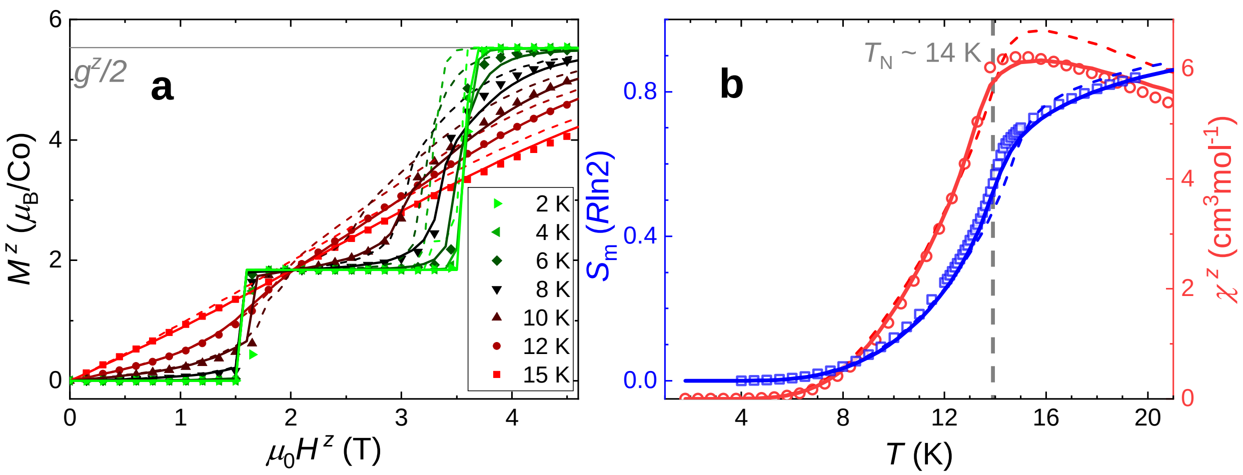

Supplementary Fig. 6 | Monte Carlo simulations of the quasi-equilibrium-state magnetism.
a Quasi-equilibrium magnetization of α-CoV_2O_6 (scatters), extracted from the data presented in Supplementary Fig. 4d. The dashed lines show the calculations using model no. 1 [5], while the solid lines represent the combined fit of the experimental data in model no. 2 (see Supplementary Tab. 1). **b** Magnetic entropy (S_m = $\int_{4\mathrm{K}}^{T} \frac{C_p(\mathrm{Co})-C_p(\mathrm{Zn})}{T'} \mathrm{d}T'$, where $C_p(\mathrm{Co})$ and $C_p(\mathrm{Zn})$ are the zero-field specific heats measured on α-CoV_2O_6 and α-ZnV_2O_6, respectively) and dc susceptibility (measured at 1 T), along with calculations from model no. 1 (dashed lines) and model no. 2 (solid lines) for comparison.

where S_i^z represents the component of the magnetic moment along the z axis on the ith site, $g^z \sim 11.04$ is the g factor determined from the magnetization (see Supplementary Fig. 6a), and the couplings beyond fourth-nearest neighbors are neglected based on the DFT calculation, which shows $|J_n| \leq 0.02|J_0|$ for $n \geq 4$ [5]. To balance computational costs and potential finite-size effects, we initially conducted standard Metropolis MC simulations above 1.9 K on a 1,296-site cluster with periodic boundary conditions (PBC). The cluster had dimensions N_a = 6, N_b = 18, N_c = 6, where N_a, N_b, and N_c represent the numbers of unit cells along the a, b, and c axes, respectively, with each unit cell containing two Co^{2+} spins. We computed the average values of thermodynamic observables at intervals of 4 Monte Carlo steps (MCS), after discarding the first 30,000 MCS for equilibration, and up to 50,000 MCS. By fitting the quasi-equilibrium magnetization above 1.9 K (see Supplementary Fig. 6a), we obtained the fully refined Hamiltonian parameters by minimizing the standard deviation σ_d (see Supplementary Tab. 1 for model no. 2). Supplementary Fig. 6 shows that the refined model no. 2, with the significantly reduced σ_d, reproduces the quasi-equilibrium magnetization, susceptibility, and entropy better than the previously reported model no. 1. Therefore, we propose that the effective spin-1/2 Hamiltonian of model no. 2 (see Supplementary Tab. 1) accurately describes the low-T magnetism of α-CoV_2O_6, without the need for any additional parameter tuning. This Hamiltonian gives rise

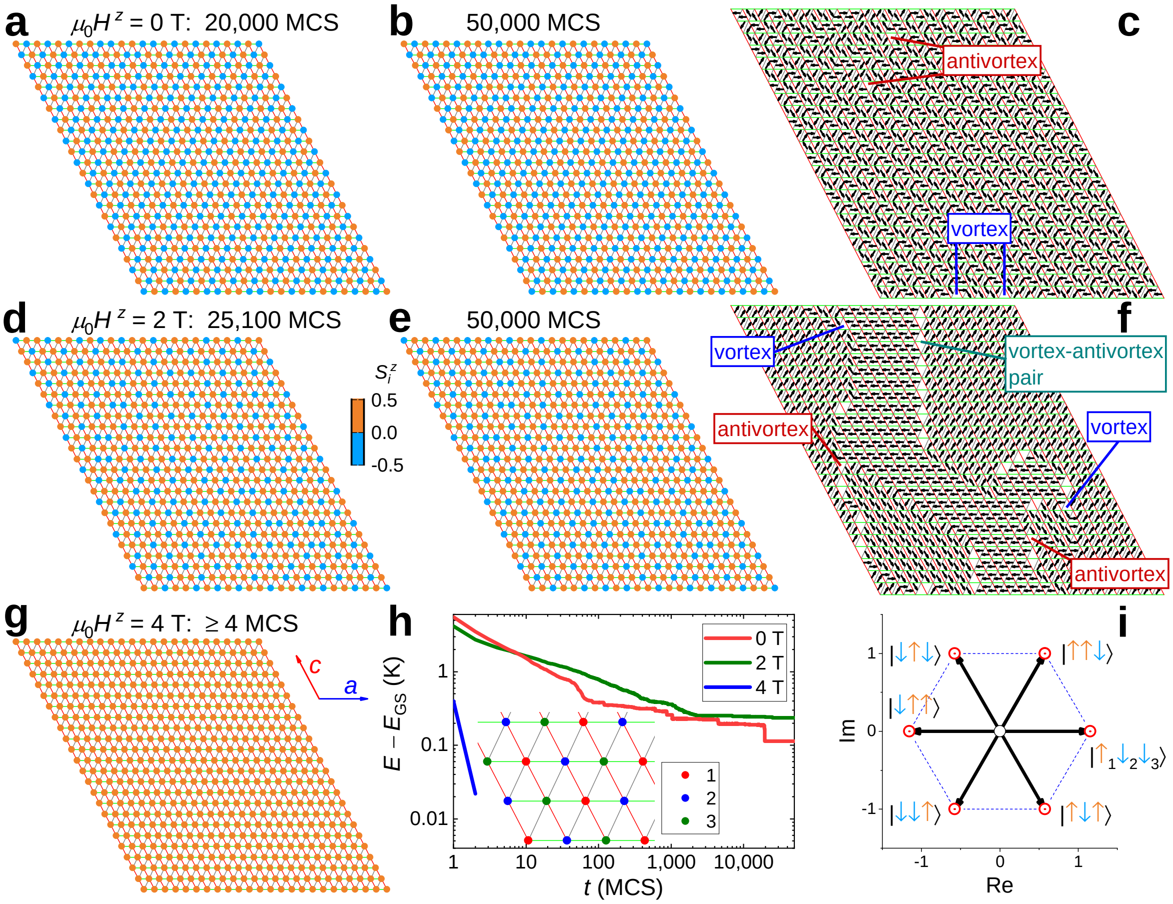

Supplementary Fig. 7 | Monte Carlo simulations using the original 3D Hamiltonian of α-CoV_2O_6 in zero transverse field. a-g Spin states calculated with external fields applied along the z (Ising) axis, specifically $\mu_0 H^z$ = 0 T (**a**-**c**), 2 T (**d**-**f**), and 4 T (**g**). After $\sim$ 400 Monte Carlo steps (MCS), spin states become independent of the site index along the b (chain) axis, indicating alignment of all Ising spins in each chain. There exist no differences between **a** and **b**, as well as between **d** and **e**. The spin state maps to a complex pseudospin field ψ_j on the triangular plaquettes ($\{1, 2, 3\}$, defined in the inset of **h**) in **c** and **f**, revealing topological features. Vortices and antivortices at defect plaquettes with ψ_j = 0 can be identified by the clockwise and anticlockwise winding of ψ_j along a closed clockwise path. **h** The time (in MCS) dependence of the residual energy per site ($E - E_{\mathrm{GS}}$). **i** The jth plaquette in the triangle lattice (see inset of **h**) has a complex pseudospin, $\psi_j = 2(\langle S^z_{1\in j}\rangle + \langle S^z_{2\in j}\rangle e^{i2\pi/3} + \langle S^z_{3\in j}\rangle e^{i4\pi/3})/\sqrt{3}$. The Monte Carlo calculations were conducted using the 28,800-site cluster with periodic boundary conditions, and were initialized with the same random state at 1.4 K, running for up to 50,000 MCS.

to three well-defined ground states under different conditions: (1) When $|H^z|$ is less than H^z_{c1}, the ground phase is the stripe AF state (see Supplementary Fig. 5c) with an energy per site of $E_{\mathrm{GS}} = \frac{J_0}{4} - \frac{J_1}{2} - \frac{J_2}{4} + \frac{J_3}{2}$ (~ -11.76 K). (2) When $H^z_{c1} < |H^z| < H^z_{c2}$, the system enters the one-third magnetization plateau state (see Supplementary Fig. 5d) with $E_{\mathrm{GS}} = \frac{J_0}{4} - \frac{J_1}{6} - \frac{J_2}{12} - \frac{J_3}{6} - \frac{\mu_0\mu_{\mathrm{B}}|H^z|g^z}{6}$. (3) When $|H^z|$ is greater than

H^z_{c2}, the spin system becomes fully polarized along the z axis with $E_{GS} = \frac{J_0}{4} + \frac{J_1}{2} + \frac{J_2}{4} + \frac{J_3}{2} - \frac{\mu_0\mu_B|H^z|g^z}{2}$. The critical longitudinal fields are given by $\mu_0 H^z_{c1} = \frac{2J_1+J_2-4J_3}{\mu_B g^z}$ ($\sim$ 1.5 T) and $\mu_0 H^z_{c2} = \frac{2J_1+J_2+2J_3}{\mu_B g^z}$ ($\sim$ 3.6 T).

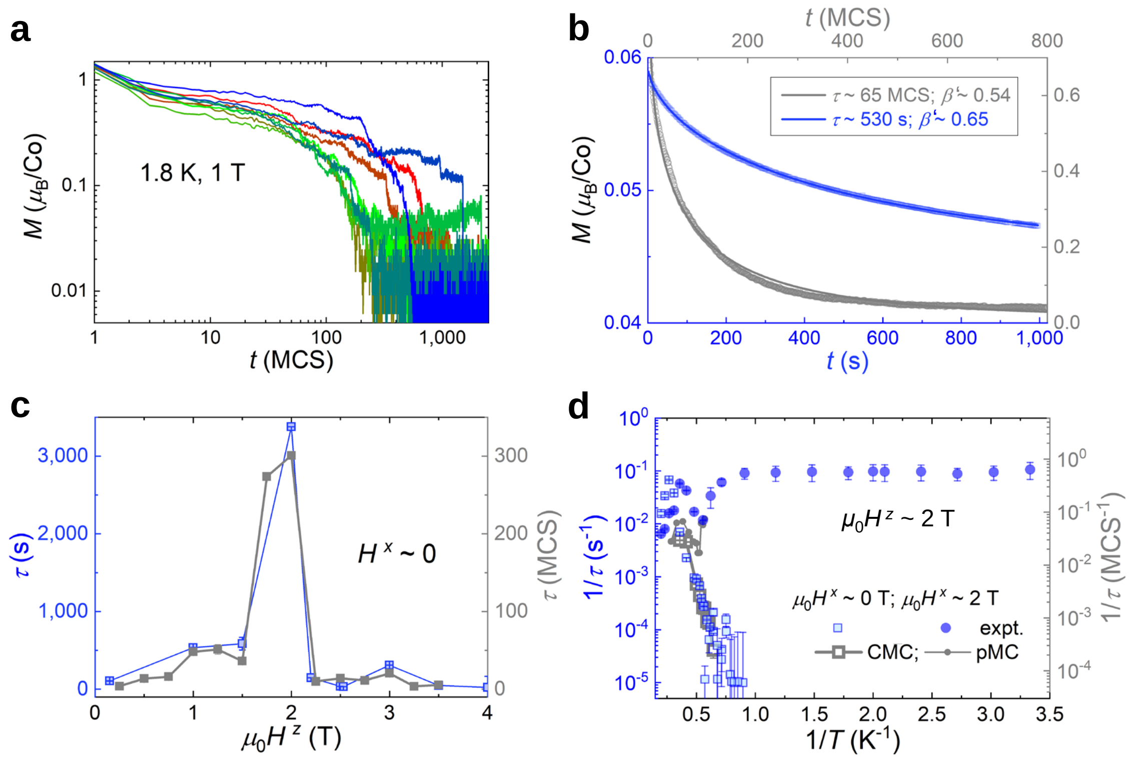

Supplementary Fig. 8 | Comparing magnetic dynamics in experiment and theory. a Magnetization (M) of 9 randomly selected samples as a function of Monte Carlo step (MCS), calculated at T = 1.8 K and $\mu_0 H^z$ = 1 T. **b** Time (t) dependence of M measured at T = 1.8 K and $\mu_0 H^z$ = 1 T (blue scatters). We collected magnetization data at t = 0, as soon as $\mu_0 H^z$ had stabilized at the set value from -4.2 T with a ramp rate of 10 mT/s. The MC magnetization (gray scatters) was calculated on the 3,600-site cluster using random initial states by evaluating over 50 independent samples. The blue and gray lines present the stretched-exponential fits to the experimental and calculated data respectively, $M(t) = (M_0 - M_\infty)\exp[-(t/\tau)^{\beta'}] + M_\infty$, where τ is the relaxation time, β' is the stretching exponent, M_0 and M_∞ are the initial and final magnetization respectively. **c** Longitudinal field ($\mu_0 H^z$) dependence of measured (blue) and calculated (gray) relaxation times at 1.8 K and $H^x \sim 0$. **d** Relaxation rates at the longitudinal magnetic field of $\mu_0 H^z \sim 2$ T, and at transverse magnetic fields of $\mu_0 H^x \sim 0$ and 2 T. The gay lines present the corresponding classical Monte Carlo (CMC) and perturbative Monte Carlo (pMC) calculations in **c** and **d**. Error bars, 1σ s.e., and the calculated MCS dependencies of energy were used to extract τ and $1/\tau$, in **c** and **d**.

To simulate the magnetic dynamics of α-CoV_2O_6 at low temperatures, we performed MC calculations using model no. 2 on a larger cluster of 28,800 sites with PBC. The cluster had dimensions of N_a = 12, N_b = 50, and N_c = 24, and we ran simulations for up to 50,000 MCS, as depicted in Supplementary Fig. 7.

We calculated the fictitious "time" (in MCS) dependence [13, 14] of magnetization on another 3,600-site cluster (with PBC and dimensions of N_a = 3, N_b = 100, and N_c = 6) using random initial states for up to 5,000 MCS, and evaluated over 50 independent samples, in Supplementary Fig. 8. Based on a comparison with experimental data (Supplementary Fig. 8c, d), we estimated that 1 MCS corresponds to approximately 10 s.

Based on the classical MC simulations described above, the low-T ($T \ll T_{\rm N}$) magnetic properties of α-CoV_2O_6 can be summarized as follows: (i) After some relaxation times, the correlation length along the chain (b axis) is much larger than that along the triangular plane (ac plane) at $\mu_0 H^z$ = 0 and 2 T (see Supplementary Fig. 7), which is attributed to the dominant intrachain FM coupling $J_0 \sim -30$ K. (ii) At low temperatures, the presence of low-energy topological excitations, such as vortices, antivortices, and vortex-antivortex pairs, may effectively confine domain walls, leading to remarkably long lifetimes ($\geq$ 20,000 MCS $\sim$ 60 hours). Our MC simulations indicate that introducing structural defects to confine magnetic domain walls is unnecessary, as good agreement with experimental data is achieved without any structural disorder (Supplementary Fig. 8). (iii) After 10 MCS, the density of excitations (see Supplementary Fig. 7c, f) and the residual energy ($E - E_{\rm GS}$, see Supplementary Fig. 7h) are typically higher at $\mu_0 H^z$ = 2 T compared to 0 T. The relaxation time exhibits a maximum at $\mu_0 H^z \sim$ 2 T, consistent with the experimental results (see Supplementary Fig. 8c). In sharp contrast, at $\mu_0 H^z$ = 4 T the spin system quickly relaxes to its ground state after only 3-4 MCS (Supplementary Fig. 7h). This suggests that the interchain spin frustration plays an important role in the extremely slow dynamics observed at low temperatures. (iv) The relaxation processes of different samples last for different times (see Supplementary Fig. 8a), leading to a distribution of the relaxation times (τ) and thus a stretched-exponential relaxation behavior [15] (e.g., see Supplementary Fig. 8b).

Supplementary Note 4. Monte Carlo simulations with small transverse fields

In zero transverse field, the Ising spin Hamiltonian of α-CoV_2O_6 (Supplementary Eq. (7)) commutes with S_i^z operators, allowing classical MC simulations on large clusters to reproduce the low-T magnetic behaviors (Supplementary Note 3). The nonzero transverse field terms, $\mathcal{H}_{\rm TF} = -\Gamma \sum_i S_i^x$, do not commute with the S_i^z operators, resulting in quantum tunnelling between states with $S_i^z = \pm 1/2$. However, this makes the computational cost much higher.

We express the Hamiltonian of Supplementary Eq. (7) as $\mathcal{H}$ = $\mathcal{H}_0$+$\mathcal{H}_{\rm TF}$, where $\mathcal{H}_0$ represents the classical Ising part. In the limit of $\beta\Gamma^2/\mid J_0 \mid\ll 1$, where $\beta \equiv 1/(k_{\rm B}T)$, we can derive an effective classical Hamiltonian $\mathcal{H}_{\rm eff}(\psi)$ as a function of the state $|\psi\rangle$, such that $e^{-\beta\mathcal{H}_{\rm eff}(\psi)} = \langle\psi|e^{-\beta\mathcal{H}}|\psi\rangle$ [16, 17]. This

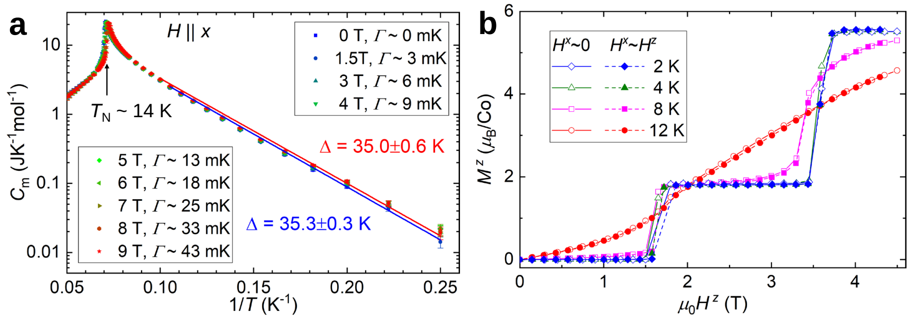

Supplementary Fig. 9 | Quasi-equilibrium-state thermodynamic properties of α-CoV_2O_6 measured with and without applied transverse fields. a Temperature dependence of specific heat (C_m) measured under various small transverse fields. Used $C_\mathrm{m} \sim \exp(-\Delta/T)$, the gaps were fit to Δ = 35.3 ± 0.3 K and 35.0 ± 0.6 K at $\mu_0 H^x$ = 0 T and 9 T, respectively, within the temperature range of 4-10 K. Alternatively, a power-law function can be used, but it only provides a good fit within a narrow range of 4-6 K, and the resulting power-law exponent of 6.4 ± 0.2 is unreasonably large. Error bars, 1σ s.e. **b** Magnetization as a function of longitudinal field.

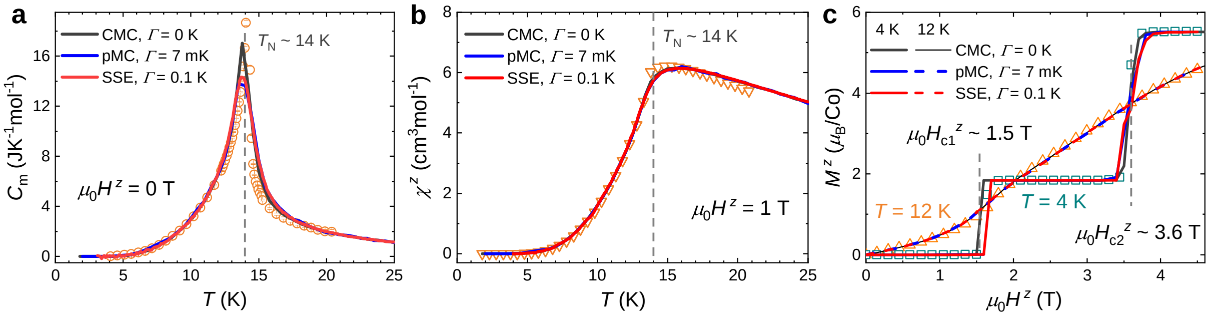

Supplementary Fig. 10 | Simulations of quasi-equilibrium-state thermodynamic properties for α-CoV_2O_6 at various small transverse fields (Γ). a magnetic specific heat, **b** susceptibility, and **c** magnetization calculated using the original 3D Hamiltonian. Methods used include classical Monte Carlo (CMC) at Γ = 0 K, perturbative Monte Carlo (pMC) at Γ = 7 mK, and stochastic series expansion (SSE) quantum Monte Carlo at Γ = 0.1 K. Experimental data are presented as scatter plots for comparison. The same set of refined interaction parameters (as described in Supplementary Tab. 1) is used. The weak Γ has little impact on these quasi-equilibrium-state thermodynamic properties, in excellent agreement with experimental results (refer to Supplementary Fig. 9). Error bars, 1σ s.e.

effective Hamiltonian is formulated as

$$\mathcal{H}_\mathrm{eff} = \mathcal{H}_0 + \beta\Gamma^2 \sum_i [2S_i^z F_1(\beta h_\mathrm{i}) - F_0(\beta h_\mathrm{i})]/4, \tag{8}$$

where h_i = $\mu_0\mu_\mathrm{B} H^z g^z - J_0 \sum_{\langle i_0\rangle} S_{i_0}^z - J_1 \sum_{\langle i_1\rangle} S_{i_1}^z - J_2 \sum_{\langle i_2\rangle} S_{i_2}^z - J_3 \sum_{\langle i_3\rangle} S_{i_3}^z$ is the local field at the ith

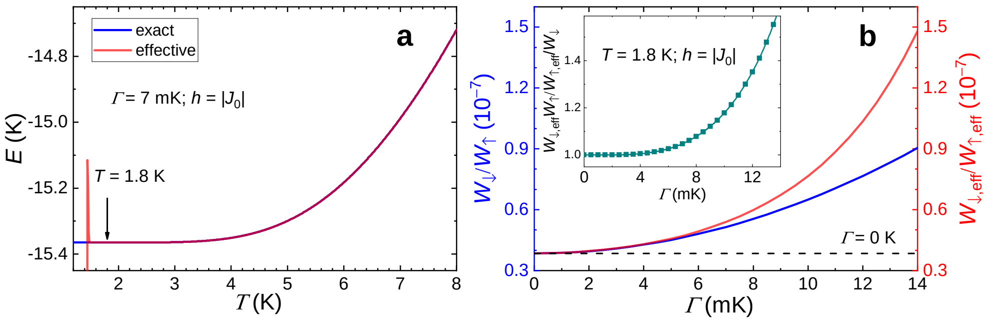

Supplementary Fig. 11 | Calculations for the single-site molecular-field model. a, **b** Equilibrium-state energies (E) and density matrix ratios ($W_{\downarrow}/W_{\uparrow}$ and $W_{\downarrow,\text{eff}}/W_{\uparrow,\text{eff}}$) calculated using the exact Hamiltonian $\mathcal{H}_{\text{s}} = -hS^z - \mathit{\Gamma} S^x$ and the effective Hamiltonian $\mathcal{H}_{\text{eff}} = -hS^z + \beta\mathit{\Gamma}^2(2S^z F_1(\beta h) - F_0(\beta h))/4$, employing a cluster mean field theory. Here, $F_0(x) \equiv \frac{\cosh(x)-1}{x^2}$ and $F_1(x) \equiv \frac{\sinh(x)-x}{x^2}$. Inset of b displays $W_{\downarrow,\text{eff}}W_{\uparrow}/W_{\uparrow,\text{eff}}/W_{\downarrow}$.

site. The functions $F_0(x)$ and $F_1(x)$ are defined as

$$F_0(x) \equiv \frac{\cosh(x)-1}{x^2}, F_1(x) \equiv \frac{\sinh(x)-x}{x^2}. \tag{9}$$

Firstly, to validate the pMC method, we examine calculated equilibrium-state thermodynamic properties as a function of temperature, following previous studies [17]. Due to the extremely long relaxation times in the low transverse field at low temperatures ($\sim$ 1.8 K) using the original spin Hamiltonian of α-CoV_2O_6, it is challenging to compute the fully equilibrium-state thermodynamic properties. In Supplementary Fig. 10, we present quasi-equilibrium-state thermodynamic properties calculated using pMC ($\mathit{\Gamma}$ = 7 mK) and classical Monte Carlo (CMC, $\mathit{\Gamma}$ = 0 mK) methods down to 2 K. These results overlap and exhibit good agreement with experimental data. Furthermore, we employed the single-site molecular-field model to calculate the fully equilibrium-state energy, providing a means to validate the perturbation theory. As illustrated in Supplementary Fig. 11a, the equilibrium-state energies calculated from the effective and exact Hamiltonians perfectly overlap above $\sim$ 1.8 K.

Secondly, we further calculated the exact and perturbative/effective density elements based on the same molecular-field model. The spin-up exact density element is computed as $W_{\uparrow} = \langle\uparrow|\exp(-\beta\mathcal{H}_{\text{s}})|\uparrow\rangle = \sum_{j1=1,2}\sum_{j2=1,2}\langle\uparrow|\psi_{j_1}\rangle\langle\psi_{j_1}|\exp(-\beta\mathcal{H}_{\text{s}})|\psi_{j_2}\rangle\langle\psi_{j_2}|\uparrow\rangle$, where $\langle\psi_{j_1}|\exp(-\beta\mathcal{H}_{\text{s}})|\psi_{j_2}\rangle = \delta_{j_1,j_2}\exp(-\beta E_{j_1})$, and E_1, E_2, $|\psi_1\rangle$, $|\psi_2\rangle$ are the eigenenergies and eigenstates of $\mathcal{H}_{\text{s}} = -hS^z - \mathit{\Gamma} S^x$. Here, $h \sim |J_0|$ represents the mean field. Similarly, we obtain $W_{\downarrow}$. On the other hand, the density element can be expressed in terms of a cumulant expansion, $W_{\uparrow} = \exp[-\beta\langle\uparrow|\mathcal{H}_{\text{s}}|\uparrow\rangle + \Sigma_{n=2}^{\infty}\frac{(-\beta)^n}{n!}\langle\uparrow|(\mathcal{H}_{\text{s}} - \langle\uparrow|\mathcal{H}_{\text{s}}|\uparrow\rangle)^n|\uparrow\rangle]$ [17]. Typically, one keeps to the lowest order $O(\mathit{\Gamma}^2)$, $\langle\uparrow|(\mathcal{H}_{\text{s}} - \langle\uparrow|\mathcal{H}_{\text{s}}|\uparrow\rangle)^n|\uparrow\rangle \sim \mathit{\Gamma}^2 h^{n-2}/4$, and obtains the perturba-

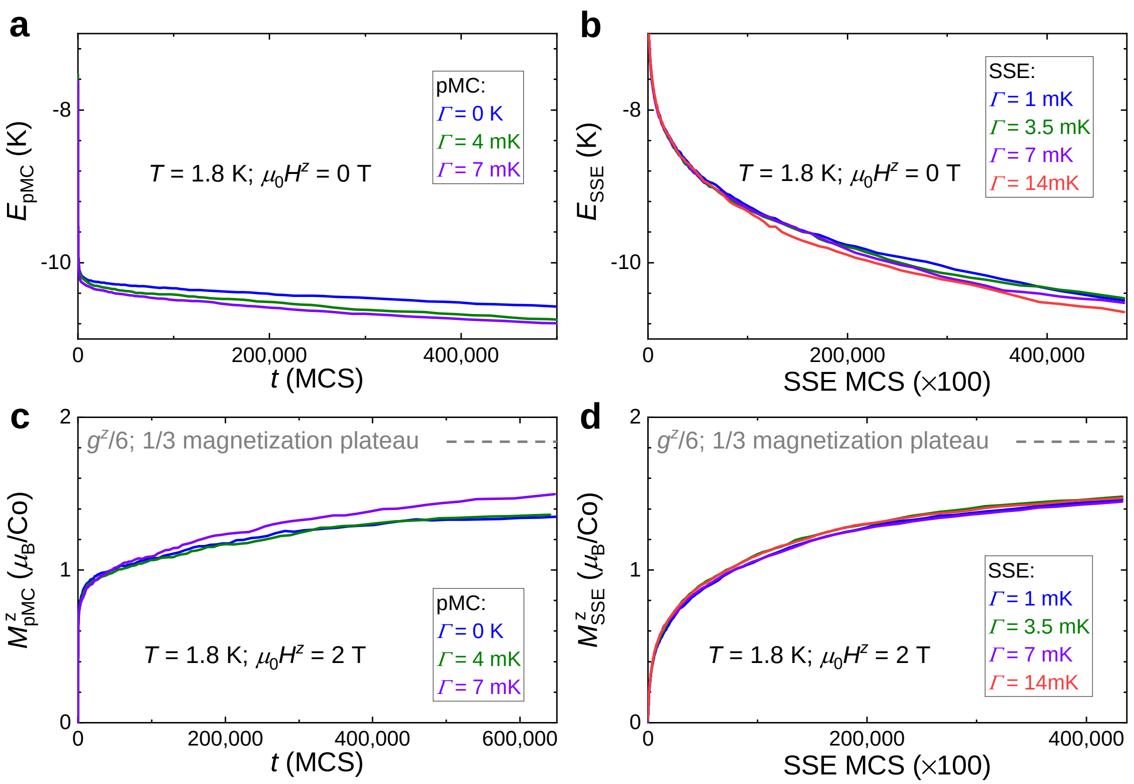

Supplementary Fig. 12 | Comparison of pMC and SSE simulations at 1.8 K. a, **b** Monte Carlo step (MCS) dependence of the energy per site calculated at zero longitudinal magnetic field ($\mu_0 H^z = 0$ T), $E_{\rm pMC}$ and $E_{\rm SSE}$. **c**, **d** MCS dependence of the longitudinal magnetization at $\mu_0 H^z = 2$ T, $M^z_{\rm pMC}$ and $M^z_{\rm SSE}$. The original spin Hamiltonian of α-CoV_2O_6 is used. The perturbative Monte Carlo (pMC) datasets are averaged over 50 independent samples. The pMC method was employed on a 12×12×24 cluster, with periodic boundary conditions. The simulations started with the fully-inversely-polarized state at $\mu_0 H^z = -4.2$ T. We gradually raised $\mu_0 H^z$ by 0.1 T at each MCS until reaching the target field and then simulated the relaxation process in subsequent MCS. For details regarding the stochastic series expansion (SSE) simulations, please refer to the main text. Please note that the classical and quantum update algorithms differ significantly.

tive density element $W_{\uparrow,\rm eff} = \exp[\beta h/2 - \beta^2 \Gamma^2 [F_1(\beta h) - F_0(\beta h)]/4] = \langle\uparrow|\exp(-\beta\mathcal{H}_{\rm eff})|\uparrow\rangle$. Here, $\mathcal{H}_{\rm eff} = -hS^z + \beta\Gamma^2[2S^z F_1(\beta h) - F_0(\beta h)]/4$. Similarly, we obtain $W_{\downarrow,\rm eff} = \exp[-\beta h/2 + \beta^2\Gamma^2[F_1(\beta h) + F_0(\beta h)]/4]$. The weight ratios calculated with the exact ($W_\downarrow/W_\uparrow$) and effective ($W_{\downarrow,\rm eff}/W_{\uparrow,\rm eff}$) models are shown in Supplementary Fig. 11b. Clearly, both the exact $W_\downarrow/W_\uparrow$ and effective $W_{\downarrow,\rm eff}/W_{\uparrow,\rm eff}$ are profoundly enhanced by a tiny transverse field Γ, supporting the quantum tunneling effect of Γ at 1.8 K. Moreover, the difference between $W_\downarrow/W_\uparrow$ and $W_{\downarrow,\rm eff}/W_{\uparrow,\rm eff}$ is not significant at $\Gamma \leq 7$ mK. Our magnetization experiments were conducted at $\Gamma \leq 7$ mK (please refer to the main text) with $1 \leq W_{\downarrow,\rm eff}W_\uparrow/W_{\uparrow,\rm eff}/W_\downarrow \leq 1.047$ at 1.8 K (see inset of Supplementary Fig. 11b), thereby supporting the validation of the pMC method. At $\Gamma = 7$ mK and

T = 1.8 K, the magnetization decreases caused by $\mathit{\Gamma}$ (and T) are calculated, $\mathit{\Delta} m_{\mathit{\Gamma}}$ = $1-\langle m\rangle$ = 1.03×10^{-7} and $\mathit{\Delta} m_{\mathit{\Gamma},\mathrm{eff}}$ = 1.08×10^{-7}, further supporting the validation of the pMC method. $\mathit{\Delta} M^z_{\mathit{\Gamma}} \sim \mathit{\Delta} M^z_{\mathit{\Gamma},\mathrm{eff}} \sim$ $1\times10^{-6}\mu_B$/Co (at $\mathit{\Gamma}$ = 7 mK and T = 1.8 K) suggests that the small transverse field has a negligible effect on the equilibrium-state magnetization, where $\mathit{\Delta} M^z_{\mathit{\Gamma}} = g\mathit{\Delta} m_{\mathit{\Gamma}}/2$ and $\mathit{\Delta} M^z_{\mathit{\Gamma},\mathrm{eff}} = g\mathit{\Delta} m_{\mathit{\Gamma},\mathrm{eff}}/2$.

At T = 1.8 K, the energy decrease caused by $\mathit{\Gamma}$ = 7 mK can be computed using the effective classical Hamiltonian, $\mathit{\Delta} E_{\mathit{\Gamma},\mathrm{eff}} = -\beta\mathit{\Gamma}^2[F_1(\beta h) - F_0(\beta h)]/4 \sim 3.8\times10^{-7}$ K. The exact decrease in the ground energy caused by $\mathit{\Gamma}$ = 7 mK is $\mathit{\Delta} E_{\mathit{\Gamma}} \sim \sqrt{\mathit{\Gamma}^2 + h^2}/2 - h/2 \sim 4.0\times10^{-7}$ K ($\sim \mathit{\Delta} E_{\mathit{\Gamma},\mathrm{eff}}$). In the limit of $\beta h \to \infty$ and $\mathit{\Gamma}/h \to 0$ (with $\mathit{\Gamma}/h \leq 2.3\times10^{-4}$ in our case), both $\mathit{\Delta} E_{\mathit{\Gamma},\mathrm{eff}}$ and $\mathit{\Delta} E_{\mathit{\Gamma}}$ converge to the same value of $\mathit{\Gamma}^2/h/4$ ($\sim 4.0\times10^{-7}$ K at $\mathit{\Gamma}$ = 7 mK), confirming the validation of the perturbation theory. Clearly, the observed decrease in energy per site caused by the tiny transverse field, $>$ 0.2 K at large MCS, cannot be attributed to the equilibrium-state energy decrease or the perturbation theory itself. Therefore, the substantial energy decrease caused by the transverse field, significantly larger than $\sqrt{\mathit{\Gamma}^2 + h^2}/2 - h/2$, should be attributed to the many-body quantum annealing effect of $\mathit{\Gamma}$.

Finally, despite distinct differences in the update algorithms and cluster sizes, our stochastic series expansion (SSE) quantum Monte Carlo simulations (please refer to the main text) for small transverse fields are in rough agreement with the pMC results in both the quasi-equilibrium-state thermodynamic properties (Supplementary Fig. 10) and out-of-equilibrium behaviors at 1.8 K (Supplementary Fig. 12). However, for larger transverse fields or lower temperatures, the pMC method may result in unphysical ground states, making it unreliable [16, 17].

Supplementary Note 5. Technical details of Faraday force magnetometer and heat transport measurements

Below 2 K, the magnetization (M, in μ_B/Co) of α-CoV_2O_6 (single crystals, $\sim$ 1 mg) was measured using a high-resolution Faraday force magnetometer in a ^{3}He-^{4}He dilution refrigerator (KELMX-400, Oxford Instruments) [18, 19]. The applied main magnetic fields (H) and field gradients ($\mathrm{d}H/\mathrm{d}l$) were generated by the superconducting coils (in INTA-LLD-S12/14, Oxford Instruments), and the electrical capacitance was measured using a digital capacitance bridge (AH-2500A, Andeen-Hagerling, Inc.) with the three-terminal method. The change in the inverse electrical capacitance is given by,

$$\Delta(\frac{1}{C}) = \frac{\Delta d}{\epsilon_0 A} = \frac{\mu_0 N_{\mathrm{Co}}\mu_\mathrm{B} M \mathrm{d}H/\mathrm{d}l}{k_{\mathrm{eff}}\epsilon_0 A}, \tag{10}$$

where ϵ_0 represents the electric permittivity of vacuum, A is the area of the top (smaller) plate, Δd denotes the variation in the gap, k_{eff} represents the effective spring coefficient in Hook's law (see Supplementary Fig. 13a), and N_{Co} the number of Co. Therefore, we obtained the magnetization by measuring the inverse

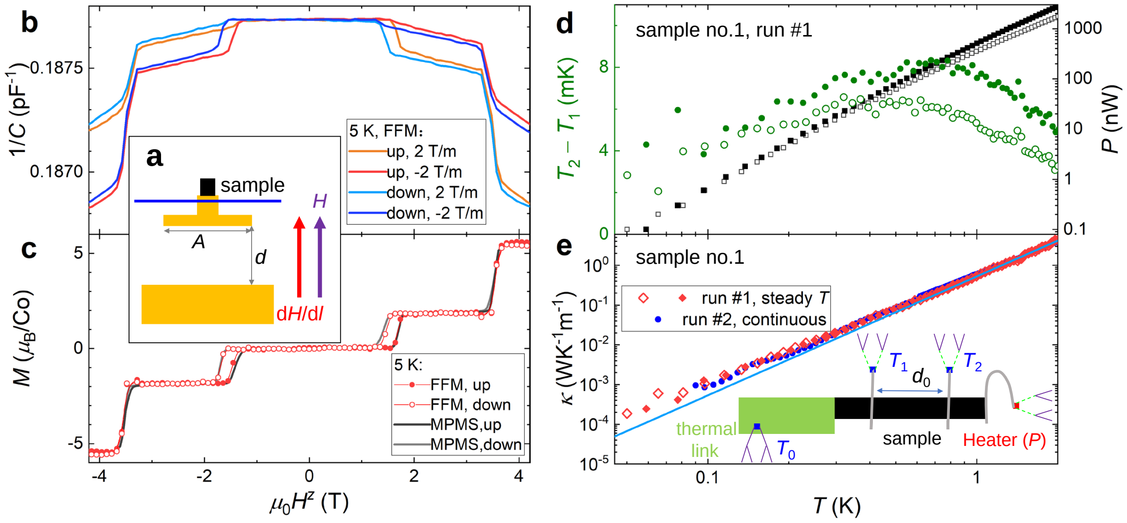

Supplementary Fig. 13 | Faraday force magnetometer and heat transport measurements on single crystals of α-CoV_2O_6. **a** Schematic diagram of the Faraday force magnetometer (FFM). **b** Inverse electrical capacitance measured by sweeping the magnetic field (applied along the z axis) up and down at different magnetic field gradients at 5 K. **c** Field dependence of magnetization measured by FFM and MPMS at 5 K. **d** The heating power applied to the sample and the resulting temperature difference between the two thermometers ($T_2 - T_1$, see inset of **e**). **e** Thermal conductivity data measured in steady-T and continuous-T sweep modes (run #1 and #2). A power-law fit to the run #1 data is represented by the line, $\kappa = \gamma T^{\alpha'}$, where α' = 2.99(2) and γ = 0.524(4) $WK^{-4}m^{-1}$. The inset illustrates a schematic diagram of the experimental setup employed for thermal conductivity measurements.

electrical capacitance (see Supplementary Fig. 13b). By scaling the results to the magnetization measured by MPMS (see Supplementary Fig. 13c), we got $k_{\rm eff} \sim 3.4 \times 10^3$ N/m at low temperatures, slightly higher than the value of $\sim 2.5 \times 10^3$ N/m measured at room temperature.

The low-temperature (down to $\sim$ 50 mK) thermal conductivity (κ, in $WK^{-1}m^{-1}$) measurements were conducted using a standard four-wire steady-state method on three single crystals of α-CoV_2O_6 in the dilution refrigerator [20]. Two samples have roughly rectangular shapes of $0.11\times0.45\times1.0$ mm^3 (sample no. 1) and $0.11\times0.56\times1.2$ mm^3 (sample no. 3), with the long edges along the b (chain) axis, whereas the sample no. 2 has dimensions of $0.15\times0.6\times1.6$ mm^3 with the long edge along the a axis (along the triangular plane). The silver wires of the two thermometers (T_1 and T_2) and heater (P) were attached to the surfaces of the samples with silver paint (see inset of Supplementary Fig. 13e), to let the heat flow along the spin chain (samples nos. 1,3) or along the a axis in the triangular plane (sample no. 2). These two RuO_2 chip thermometers (T_1 and T_2, RX-102A-BR, LakeShore) were *in situ* calibrated against a reference (T_0, RX-102B-RS-0.02B, LakeShore, calibrated down to 20 mK) at each applied magnetic field, by turning off

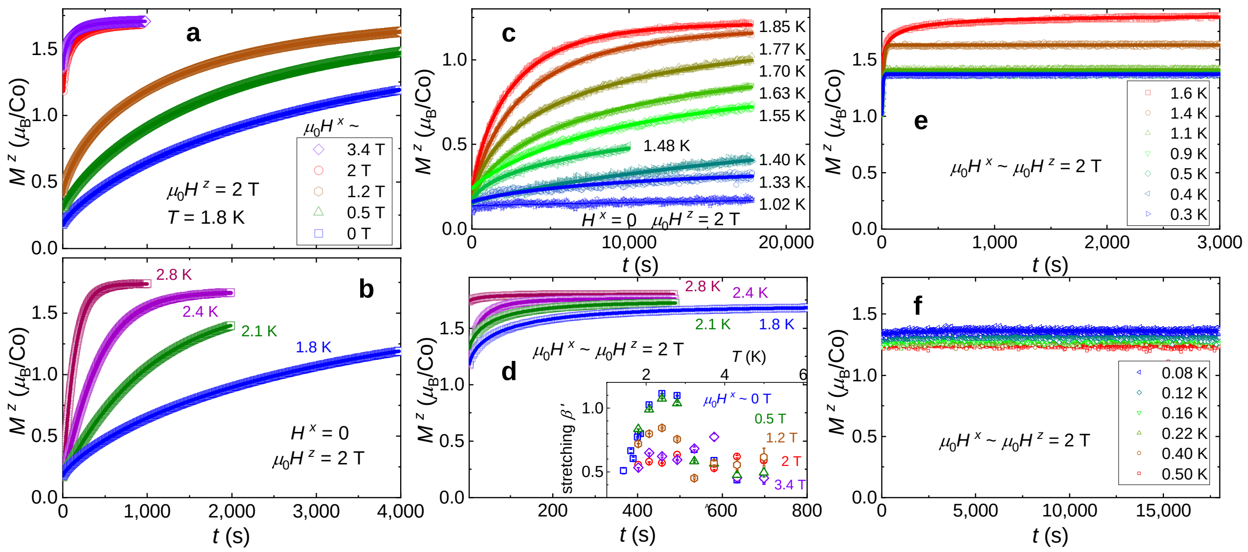

Supplementary Fig. 14 | Magnetization relaxation data of α-CoV$_2$O$_6$. a Magnetization measured at $\mu_0 H^z$ = 2 T and T = 1.8 K, with different rotation angles between the Ising (z) direction and applied field: θ = 60° ($\mu_0 H^x = \mu_0 H^z \tan\theta$ = 3.4 T), 45° ($\mu_0 H^x$ = 2 T), 30° ($\mu_0 H^x$ = 1.2 T), 15° ($\mu_0 H^x$ = 0.5 T), 0° ($\mu_0 H^x$ = 0 T). **b, c** Measurements with $\mu_0 H^z$ = 2 T and θ = 0° at various temperatures. **d-f** Relaxation data measured with $\mu_0 H^z$ = 2 T and θ = 45°. We define the moment t = 0 as $\mu_0 H^z$ reaches 2 T from -4.2 T (where all spins are fully-inversely-polarized), using a constant ramp rate of $\mu_0 \mathrm{d}H^z/\mathrm{d}t$. In panel **f**, we used a low rate of $\mu_0 \mathrm{d}H^z/\mathrm{d}t$ = 1 mT/s for $t < 0$ s to maintain the low sample temperature, whereas 10 mT/s (at $t < 0$ s) was applied in panels **a-e**. The experimental data (scatters) are shown with the colored lines representing the stretched-exponential fits, in **a-e**. The stretching exponents β' are shown in the inset of **d**. Error bars, 1σ s.e.

the heater (i.e., at P = 0). The thermal conductivity is obtained as

$$\kappa(T = \frac{T_2 + T_1}{2}) = \frac{d_0 P}{A_0(T_2 - T_1)}, \tag{11}$$

where d_0 is the distance between the two thermometers (T_1 and T_2), A_0 represents the cross-section area of the single-crystal sample, T_1 and T_2 are the measured temperatures of the thermometers, and P the measured power of the heater (see Supplementary Fig. 13d). d_0 = 0.40, 0.66, 0.50 mm, A_0 = 0.050, 0.090, 0.062 mm^2, for samples nos. 1, 2, 3 respectively, were determined using a microscope.

The final thermal conductivity is independent of the excited power P (see Supplementary Fig. 13d, e), suggesting good linear response, and is also independent of different measurement modes (Supplementary Fig. 13e). No significant sample dependence of κ_b between samples nos. 1 and 3 was observed (Supplementary Fig. 15a). As shown in Supplementary Fig. 15b κ_b is less sensitive to longitudinal magnetic fields $\mu_0 H^z$ than κ_a to transverse magnetic fields $\mu_0 H^y$ (Supplementary Fig. 15c). Here, κ_a and κ_b are the thermal conductivities measured along the a and b axes, respectively. The slight suppression of κ_b by

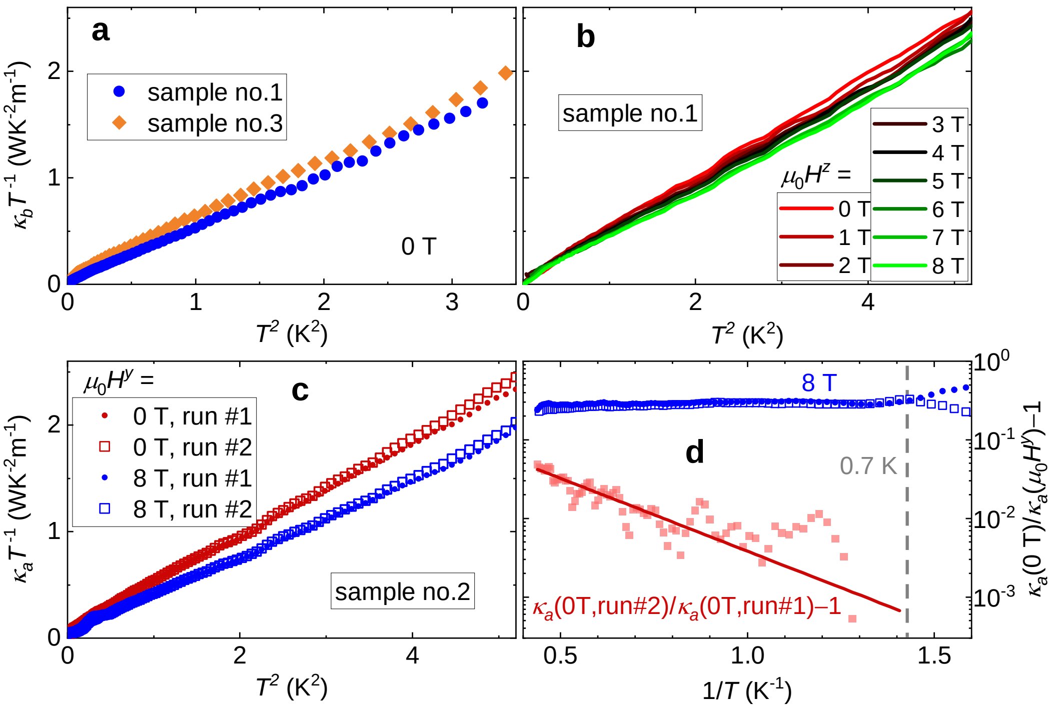

Supplementary Fig. 15 | Extended thermal conductivity data of α-CoV$_2$O$_6$. a Zero-field thermal conductivity measured along the b axis (κ_b) on two samples (nos. 1 and 3). **b** The data measured on sample no. 1 under various longitudinal magnetic fields. **c** Thermal conductivity measured along the a axis (κ_a) on sample no. 2 under transverse magnetic fields of $\mu_0 H^y$ = 0 and 8 T. We measured the data at 1 T intervals from 0 to 8 T twice, with the first measurement designated as run #1 (0 $\rightarrow$ 8 T) and the second as run #2 (0 $\rightarrow$ 8 T, again). **d** κ_a(0 T)/$\kappa_a(\mu_0 H^y)-1$. The red line displays the Arrhenius fit to the experimental data of κ_a(0 T, run #2)/κ_a(0 T, run #1)-1 above $\sim$ 0.7 K, $\propto \exp(-\Delta E/T)$, with ΔE = 4.3±0.3 K.

$\mu_0 H^z$ can be attributed to increased inelastic phonon-spin scattering, which is enhanced by closing the spin gap (see Supplementary Fig. 2c) [21]. Moreover, Supplementary Fig. 15c shows that κ_a has a weak dependence on run sequences. Long-term exploration (about 10 days) at low temperatures ($\leq$ 2.3 K) under transverse magnetic fields (up to $\mu_0 H^y$ = 8 T) only slightly increases κ_a, suggesting that the magnetic crystal is approaching its quasi-equilibrium state. In this work, we utilized the most recently measured κ_a at zero field (in run #2), which had the highest values for almost the entire temperature range (see Supplementary Fig. 15c), as the pure phonon thermal conductivity $\kappa_{\rm p}$. As explained in the main text, we used κ_a(0 T, run #2)/$\kappa_a(\mu_0 H^y)-1$ to approximate $\tau_{\rm pp}/\tau_{\rm ps}$, where $\tau_{\rm pp}^{-1}$ and $\tau_{\rm ps}^{-1}$ are the pure phonon and phonon-spin scattering rates, respectively. $\tau_{\rm pp}$ is expected to be independent of temperature and applied magnetic field below 2.3 K ($\ll$ Debye temperature of $\sim$ 160 K). Hence, κ_a(0 T, run #2)/$\kappa_a(\mu_0 H^y)-1$ is an approximate representation of the spin fluctuations around magnetic domain walls, as the system undergoes annealing

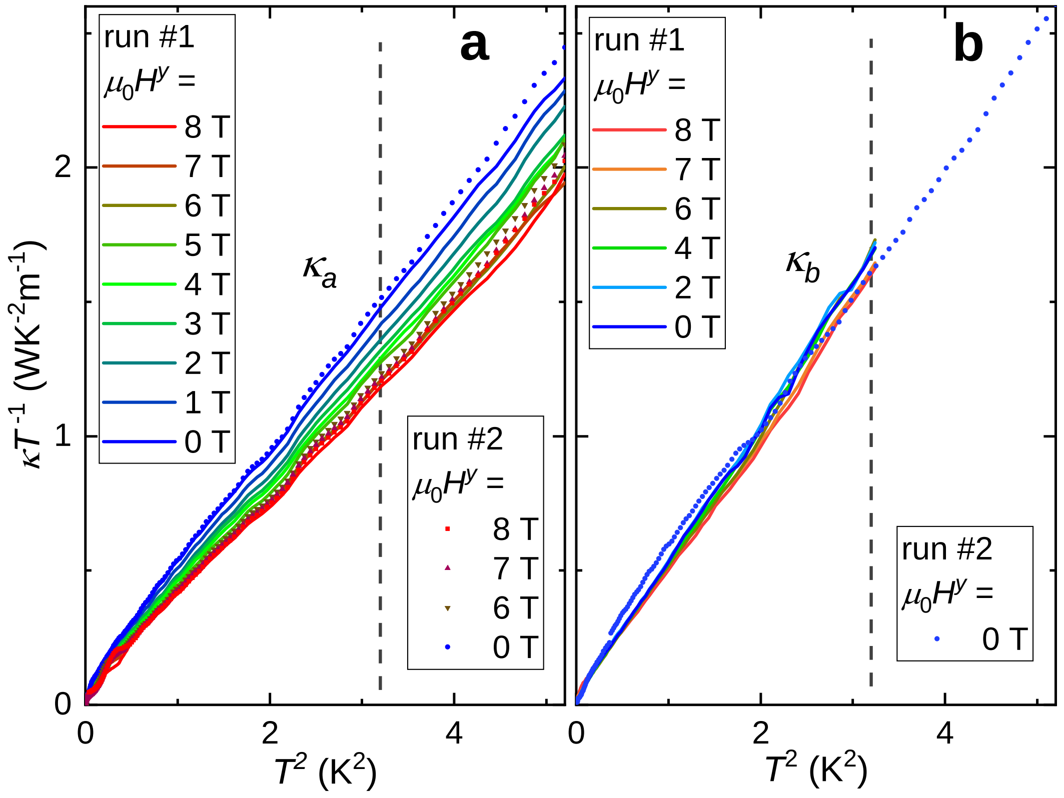

Supplementary Fig. 16 | Comparison of thermal conductivity (κ) measurements in α-CoV_2O_6 along the a (κ_a, a) and b (κ_b, b) axes under various transverse magnetic fields ($\mu_0 H^y$). In run #1, κ_a was measured between $\sim$ 0.06 and 2.3 K (measured by warming up) under steady $\mu_0 H^y$ ranging from 0 to 8 T. Each $\mu_0 H^y$ measurement took 1 day, resulting in a total duration of approximately 10 days for run #1, including cooling-down and liquid-helium-filling intervals. Following the completion of run #1, we cooled down to the base temperature of $\sim$ 0.05 K, set $\mu_0 H^y$ to 0 T, and subsequently initiated the measurements for run #2 in sequence.

by flipping spins near the domain walls. As shown in Supplementary Fig. 15d, κ_a(0 T, run $\#2$)/κ_a(0 T, run $\#1$)-1 exhibits a thermally activated Arrhenius behavior above $\sim$ 0.7 K, $\propto \exp(-\Delta E/T)$, where ΔE = 4.3±0.3 K represents the zero-field barrier energy. In contrast, κ_a(0 T, run $\#2$)/$\kappa_a(\mu_0 H^y)-1$ ($\mu_0 H^y \geq 1$ T) remains almost constant throughout the same temperature range, suggesting the emergence of quantum spin fluctuations induced by the transverse field.

The thermal conductivity (κ_a) measured at the same transverse magnetic field and temperature indeed exhibits a weak increase in run #2 compared to run #1, following a long-term exploration in $\mu_0 H^y$ (Supplementary Fig. 16a). This phenomenon is consistently observed across different transverse magnetic fields of $\mu_0 H^y$ = 0, 6, 7, 8 T (refer to Supplementary Fig. 16a). These observations potentially suggest a decrease in the concentration of magnetic domain walls and defects along the triangular plane in run #2 compared to run #1, following the prolonged annealing period. However, the suppression of κ_a by $\mu_0 H^y$ remains

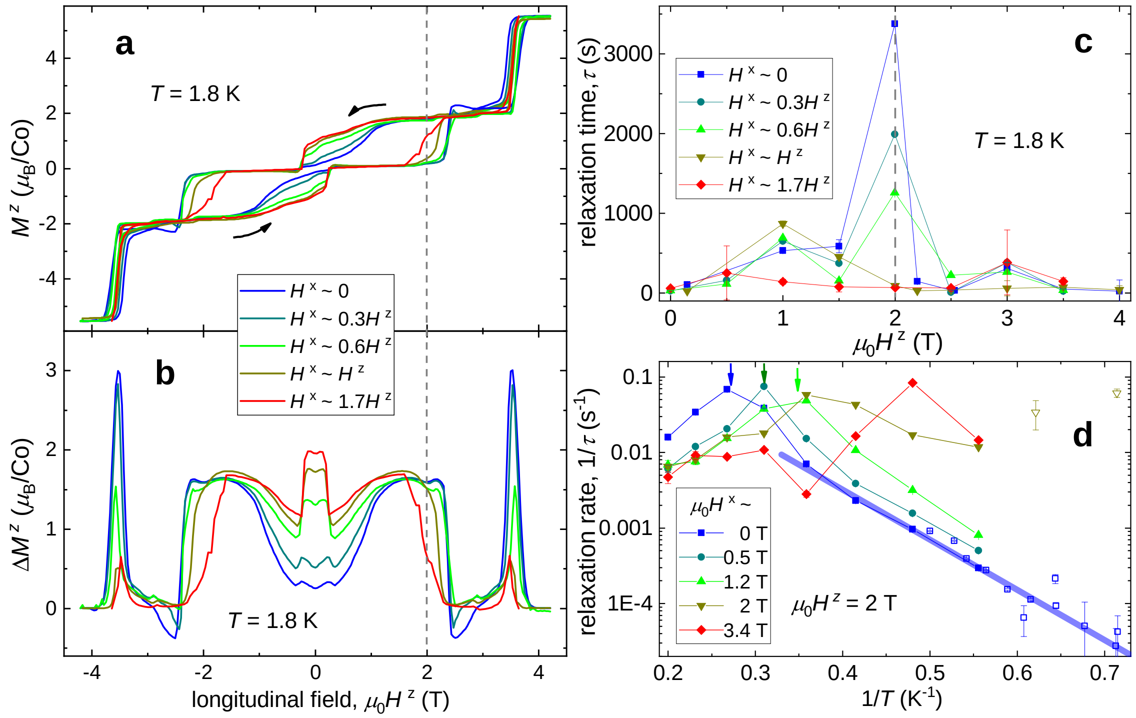

Supplementary Fig. 17 | Magnetic properties of α-CoV$_2$O$_6$ measured with $\theta \sim$ 0°, 15°, 30°, 45° and 60° in a MPMS. a M^z-H^z hysteresis loops measured at 1.8 K under different applied transverse fields (H^x) with respective to the longitudinal field (H^z), H^x = $\tan\theta H^z$. **b** The loop widths of the curves in **a**. **c** The relaxation time τ measured under H^x = $\tan\theta H^z$ at 1.8 K. **d** Inverse-temperature dependence of the relaxation rate τ^{-1} at the longitudinal field of $\mu_0 H^z$ = 2 T and various transverse magnetic field of $\mu_0 H^x \sim$ 0, 0.5, 1.2, 2, 3.4 T. θ represents the angle between the Ising (z) direction and the applied magnetic field **H**, and the colored arrows indicate the peak temperatures. The low-T data measured at $\mu_0 H^x \sim$ 0 T (hollow squares) and 2 T (hollow triangles) in a dilution refrigerator are shown for comparison. The thick blue line displays the Arrhenius behavior of $\tau^{-1} \propto \exp(-\Delta E/T)$. Error bars, 1σ s.e.

largely repeatable in run #2 (Supplementary Fig. 16a), suggesting that the magnetic imperfections do not completely disappear even after the long-term (> 1 day) annealing in $\mu_0 H^y$ (up to 8 T) between temperatures of 0.05 and 2.3 K. The annealing process of the frustrated spin system α-CoV$_2$O$_6$ towards the ground state continues even after long-term exploration at a transverse field of $\Gamma <$ 0.1 K. This implies that the ideal single-domain state is never achieved experimentally in α-CoV$_2$O$_6$ at low temperatures and in $\Gamma <$ 0.1 K. The measured magnetization (M^z) does not reach the expected value of g^z/6 = 1.8 μ_B/Co, even after a prolonged annealing in the transverse field at low temperatures ($M^z \leq$ 1.4 μ_B/Co, see Fig. 2 in the main text). Furthermore, our pMC simulations indicate that metastable states of the 3,456-site system can have extremely long lifetimes at $\Gamma \leq$ 7 mK, lasting up to > 500,000 MCS $\sim$ 50 days (see Fig. 4 in main text). Therefore, complete annealing of the quasi-infinite system of α-CoV$_2$O$_6$ in the thermodynamic limit (at Γ

< 0.1 K) would require even more time.

Supplementary Note 6. Classical-quantum crossover behavior induced by the transverse field

The loop width is progressively suppressed as the transverse magnetic field increases, at $\mu_0|H^z| > \mu_0 H^z_{c1} \sim 1.5$ T (see Supplementary Fig. 17a, b). Additionally, the transverse magnetic field gradually decreases the relaxation time at $\mu_0 H^z$ = 2 T and low temperatures (see Supplementary Fig. 17c, d). The transverse magnetic field gradually lowers the peak temperature below which the Arrhenius behavior of $\tau^{-1} \propto \exp(-\Delta E/T)$ becomes visible (see Supplementary Fig. 17d). This observed classical-quantum crossover is reminiscent of similar findings previously reported in $LiHo_xY_{1-x}F_4$ [22].

Supplementary References

[1] Z. He, J.-I. Yamaura, Y. Ueda, and W. Cheng, "CoV_2O_6 single crystals grown in a closed crucible: unusual magnetic behaviors with large anisotropy and $\frac{1}{3}$ magnetization plateau," J. Am. Chem. Soc. **131**, 7554–7555 (2009).

[2] Z. He and W. Cheng, "Magnetic phase diagram of an Ising spin-chain system α-CoV_2O_6 with 1/3 magnetization plateau," J. Magn. Magn. Mater. **362**, 27–30 (2014).

[3] C. B. Liu, Z. Z. He, S. L. Wang, M. Yang, Y. Liu, Y. J. Liu, R. Chen, H. P. Zhu, C. Dong, J. Z. Ke, Z. W. Ouyang, Z. C. Xia, and J. F. Wang, "Field-induced magnetic transitions and strong anisotropy in α-CoV_2O_6 single crystal," J. Phys.: Condens. Matter **31**, 375802 (2019).

[4] M. Lenertz, J. Alaria, D. Stoeffler, S. Colis, A. Dinia, O. Mentré, G. André, F. Porcher, and E. Suard, "Magnetic structure of ground and field-induced ordered states of low-dimensional α-CoV_2O_6: Experiment and theory," Phys. Rev. B **86**, 214428 (2012).

[5] A. Saúl, D. Vodenicarevic, and G. Radtke, "Theoretical study of the magnetic order in α-CoV_2O_6," Phys. Rev. B **87**, 024403 (2013).

[6] Y. Li, D. Adroja, R. I. Bewley, D. Voneshen, A. A. Tsirlin, P. Gegenwart, and Q. Zhang, "Crystalline electric-field randomness in the triangular lattice spin-liquid $YbMgGaO_4$," Phys. Rev. Lett. **118**, 107202 (2017).

[7] https://docs.mantidproject.org/nightly/concepts/CrystalField.html.

[8] J. Kanamori, "Theory of the magnetic properties of ferrous and cobaltous oxides, I," Prog. Theor. Phys. **17**, 177–196 (1957).

[9] J. A. Ringler, A. I. Kolesnikov, and K. A. Ross, "Single-ion properties of the transverse-field Ising model material $CoNb_2O_6$," Phys. Rev. B **105**, 224421 (2022).

[10] Y. Li, S. Bachus, H. Deng, W. Schmidt, H. Thoma, V. Hutanu, Y. Tokiwa, A. A. Tsirlin, and P. Gegenwart, "Partial up-up-down order with the continuously distributed order parameter in the triangular antiferromagnet $TmMgGaO_4$," Phys. Rev. X **10**, 011007 (2020).

[11] Y. Li, Q.-Y. Li, W. Li, T. Liu, D. J. Voneshen, P. K. Biswas, and D. Adroja, "Spin dynamics and Griffiths singularity in the random quantum Ising magnet $PrTiNbO_6$," npj Quantum Mater. **6**, 34 (2021).

[12] M. Nandi and P. Mandal, "Magnetic and magnetocaloric properties of quasi-one-dimensional Ising spin chain CoV_2O_6," J. Appl. Phys. **119**, 133904 (2016).

[13] G. E. Santoro, R. Martoňák, E. Tosatti, and R. Car, "Theory of quantum annealing of an Ising spin glass," Science **295**, 2427–2430 (2002).

[14] S. Kobayashi, H. Okano, T. Jogetsu, J. Miyamoto, and S. Mitsuda, "Domain growth kinetics in the isosceles triangular Ising antiferromagnet $CoNb_2O_6$," Phys. Rev. B **69**, 144430 (2004).

[15] P. Ocampo-Alfaro and H. Guo, "Cooling-rate dependence of the ground-state energy using microcanonical simulated annealing," Phys. Rev. E **53**, 1982–1985 (1996).

[16] R. J. Creswick, H. A. Farach, J. M. Knight, and C. P. Poole, "Monte Carlo method for the Ising model in a transverse field," Phys. Rev. B **38**, 4712–4715 (1988).

[17] S. M. A. Tabei, M. J. P. Gingras, Y.-J. Kao, and T. Yavors'kii, "Perturbative quantum Monte Carlo study of $LiHoF_4$ in a transverse magnetic field," Phys. Rev. B **78**, 184408 (2008).

[18] Y. Shimizu, Y. Kono, T. Sugiyama, S. Kittaka, Y. Shimura, A. Miyake, D. Aoki, and T. Sakakibara, "Development of high-resolution capacitive Faraday magnetometers for sub-Kelvin region," Rev. Sci. Instrum. **92**, 123908 (2021).

[19] Y. Li, S. Bachus, B. Liu, I. Radelytskyi, A. Bertin, A. Schneidewind, Y. Tokiwa, A. A. Tsirlin, and P. Gegenwart, "Rearrangement of uncorrelated valence bonds evidenced by low-energy spin excitations in $YbMgGaO_4$," Phys. Rev. Lett. **122**, 137201 (2019).

[20] X. Hong, M. Behnami, L. Yuan, B. Li, W. Brenig, B. Büchner, Y. Li, and C. Hess, "Heat transport of the kagome Heisenberg quantum spin liquid candidate $YCu_3(OH)_{6.5}Br_{2.5}$: Localized magnetic excitations and a putative spin gap," Phys. Rev. B **106**, L220406 (2022).

[21] D. Watanabe, K. Sugii, M. Shimozawa, Y. Suzuki, T. Yajima, H. Ishikawa, Z. Hiroi, T. Shibauchi, Y. Matsuda, and M. Yamashita, "Emergence of nontrivial magnetic excitations in a spin-liquid state of kagomé volborthite," Proc. Natl. Acad. Sci. **113**, 8653–8657 (2016).

[22] J. Brooke, T. F. Rosenbaum, and G. Aeppli, "Tunable quantum tunnelling of magnetic domain walls," Nature **413**, 610–613 (2001).
